# Statistics for Evaluating Pre-post Change: Relation Between Change in the Distribution Center and Change in the Individual Scores

**DOI:** 10.3389/fpsyg.2018.02696

**Published:** 2019-01-08

**Authors:** Eduardo Estrada, Emilio Ferrer, Antonio Pardo

**Affiliations:** ^1^Department of Social Psychology and Methodology, Universidad Autónoma de Madrid, Madrid, Spain; ^2^Department of Psychology, University of California, Davis, Davis, CA, United States

**Keywords:** individual reliable change, effect size estimation, assessment of change, Reliable Change Index (RCI), pre-post change

## Abstract

In a number of scientific fields, researchers need to assess whether a variable has changed between two time points. Average-based change statistics (*ABC*) such as Cohen's *d* or Hays' ω^2^ evaluate the change in the distributions' center, whereas Individual-based change statistics (*IBC*) such as the Standardized Individual Difference or the Reliable Change Index evaluate whether each case in the sample experienced a reliable change. Through an extensive simulation study we show that, contrary to what previous studies have speculated, *ABC* and *IBC* statistics are closely related. The relation can be assumed to be linear, and was found regardless of sample size, pre-post correlation, and shape of the scores' distribution, both in single group designs and in experimental designs with a control group. We encourage other researchers to use *IBC* statistics to evaluate their effect sizes because: (a) they allow the identification of cases that changed reliably; (b) they facilitate the interpretation and communication of results; and (c) they provide a straightforward evaluation of the magnitude of empirical effects while avoiding the problems of arbitrary general cutoffs.

The evaluation of change is a key goal in all sciences. In psychological, education, and medical sciences, there is a long tradition of using effect size measures both to quantify the amount of change experienced by a group across several time points, and for comparing such change in multiple groups (Cohen, 1988; Richardson, 1996; Fritz et al., 2012; Grissom and Kim, 2012; Kelley and Preacher, 2012; Pek and Flora, 2018). This paper examines the assessment of change through individuals' responses to standardized tests. Specifically, we focus on situations in which the same variable is measured at two time points in all the individuals in the sample (i.e., *Pre-post research designs*).

Pre-post designs are often used when an intervention is applied between the two time points. Whether the observed change can be attributed to the intervention or not depends on a number of factors, including whether (a) a control group exists; (b) the study is experimental, quasi-experimental or observational; (c) relevant covariates and cofounds have been adequately controlled (Fisher, 1935; Rubin, 1974; Shadish et al., 2002; Pearl, 2009; Mayer et al., 2016).

Very often, researchers want to cause a change with their interventions. Some examples are: (a) A school teacher applying a visual-spatial training program to children of ages 10–12 would want them to increase their ability (c.f. Lowrie et al., 2017); (b) Numerous programs for cognitive training are intended to increase working memory capacity—and, ultimately, general cognitive ability—in their participants (c.f. Jaeggi et al., 2008); (c) Interventions with teenagers with autism spectrum disorders typically aim at improving their interpersonal and communication skills, among others; (d) Interventions in clinical psychology typically are intended to change the clients' behavior so they can adapt better to their environment and increase their quality of life (e.g., gain social skills, control their anger or anxiety, improve their depressive symptoms, or avoid their maladaptive behaviors, among others; c.f. Muroff et al., 2014); (e) A pharmacological treatment for obesity will be successful if the patients reduce their weight (c.f. Pi-Sunyer et al., 2015)^1^.

In a pre-post research design, some criterion is needed to determine large or small change. Here we focus on *distribution based methods* (i.e., there is no external information or clinical referents, other than the test scores; Lydick and Epstein, 1993; Crosby et al., 2003; Revicki et al., 2008). These methods attempt to identify the smallest change that cannot be explained by sampling random fluctuations or by measurement error (Jacobson and Truax, 1991; Crosby et al., 2003; Bauer et al., 2004). This amount of change is usually called *statistically reliable, minimally detectable* or just *reliable change* (Maassen, 2000; Beaton et al., 2001; de Vet et al., 2006).

To detect a reliable change, two approaches can be adopted. We termed them the *average-based change* approach (ABC) and the *individual-based change* approach (IBC). The aim of ABC is to evaluate whether a group, as a whole, experienced a reliable change. In turn, the goal of IBC is to identify specific individuals who showed change. To assess ABC, researchers often use a statistic that describes the center of the distributions (often, the pre and post means), by using null hypothesis tests and effect size measures (c.f., Cohen, 1988; Fritz et al., 2012; Grissom and Kim, 2012; Pek and Flora, 2018). To assess IBC, researchers may use various indices that can be grouped under the name of *reliable change indices*. Some of these indices are based on standardization of pre-post differences, others on the standard error of measurement, and yet others on linear regression predictions (Crosby et al., 2003; Ferrer and Pardo, 2014).

The goal of this paper is twofold. First, we want to investigate the relation between ABC and IBC statistics, and to describe such a relation in mathematical terms. We show that, contrary to what other previous studies have speculated, both approaches are strongly related. Second, we attempt to draw researchers' attention to a set of tools derived from individual-based statistics. These are simple tools that can provide help in a variety of research contexts. We show how they can be used for intuitive interpretation and communication of research results, and how they can replace arbitrary cutoffs (e.g., Cohen, 1988) commonly used for deciding when an effect is “small” or “large.”

## Are the Average-Based and the Individual-Based Approaches Related?

Many studies have argued that the information provided by these two approaches is different. Below are some examples:

“Statistical methods based on the General(ized) Linear Model (…) have optimal power when individuals behave identically (…). When there exists genuine, idiosyncratic variations in the effect of a factor, (…) the effect of a factor can be significant for every individual (…) while Student and Fisher tests yield a probability close to one if the population average is small enough” (Vindras et al., 2012, p. 2).

“Statistically significant change at the group level may not be significant at the individual level (…). Mean changes for a group may be the result of few individuals with relatively large changes, or numerous individuals with relatively small changes” (Schmitt and Di Fabio, 2004, pp. 1008–1009).

Similar ideas can be found in other studies (e.g., Ottenbacher et al., 1988; Testa, 2000). Accordingly, it appears that average and individual approaches focus on *different* aspects of change, inasmuch as knowing that the center of the scores distribution changed provides no information about which particular individuals changed. Indeed, the change in the distribution center and the percentage of individual changes are calculated in very different ways.^2^

However, it is not evident whether these two approaches are completely *independent*. Rather, it is reasonable to think that the larger the displacement of distribution center, the higher the percentage of reliable individual changes. In fact, the higher the mean of the pre-post differences, the more likely it is that a pre-post difference exceeds a certain cutoff. For example, if the pre-post differences distribution is normal, the probability associated with each cutoff is known. If the mean of the differences equals zero, the probability of finding cases above 1.645 standard deviations equals 0.05. If the mean of the differences is 0.5 standard deviations above zero, the probability of finding cases above 1.645 standard deviations equals 0.13, etc. However, these probabilities are unknown when the pre-post differences distribution is *non-normal*, which is the usual case in applied contexts.

One study showed that the pre-post effect size observed (i.e., the magnitude of change in distribution center) is the main determinant of the percentage of individuals showing pre-post change (Norman et al., 2001). This simulation study revealed that the relation between effect size and percentage of change is approximately linear for effect sizes below one, with normal and moderately skewed distributions, and regardless of the cutoff to detect a change. Therefore, at least under certain conditions, the mean change can yield some information about the percentage of individual changes. A later study using empirical data found consistent results (Lemieux et al., 2007). However, these papers did not report any mathematical function to estimate the percentage of changes based on the change in the distribution center, nor did they report the fit that such a function may achieve, which would be useful to assess the quality of its estimations.

The scarcity of studies on this topic and the lack of sound conclusions suggest that more research is needed to understand the relation between the change in the distribution center and the percentage of individual changes.

## The Present Study

Our first goal in this article was to investigate the relation between ABC and IBC statistics, and to mathematically describe that relation. Specifically, we sought to: (a) investigate whether ABC and IBC are related; (b) if so, identify its shape, a mathematical function that best represents it, and the goodness of fit of such function; and (c) determine what conditions affect the nature of the relation. For this, we conducted a simulation study corresponding to two of the most common designs in the behavioral and social sciences: a “pre-post design” and a “control group pre-post design.” To our knowledge, this is the first study applying individual change indices to a pre-post design with a control group. Importantly, we studied this relation in scenarios with both normal and non-normal distributions.

Our second goal was to promote the use of individual-based statistics as a simple and useful tool for addressing important research questions. Based on our simulation results, we show that such statistics can be used to interpret research results and make decisions in applied settings.

## Methods

We simulated data for two scenarios in which the same variable is measured at two time points (e.g., before and after the intervention) for each individual within a group of participants. We generated two different pre-post research designs: *with* and *without a control group*.

Including a single group design is important because: (a) this is a common scenario in applied contexts; and (b) all indices describing the percentage of individual changes were developed for settings with a single treated group (Payne and Jones, 1957; Jacobson and Truax, 1991; Crawford et al., 1998; Hageman and Arrindell, 1999; Wyrwich et al., 1999). On the other hand, it is well known that including a control group—ideally, with random assignment—provides stronger evidence for attributing the change to the treatment (Shadish et al., 2002; Feingold, 2009).

### Simulation Conditions

**Figure 1:**
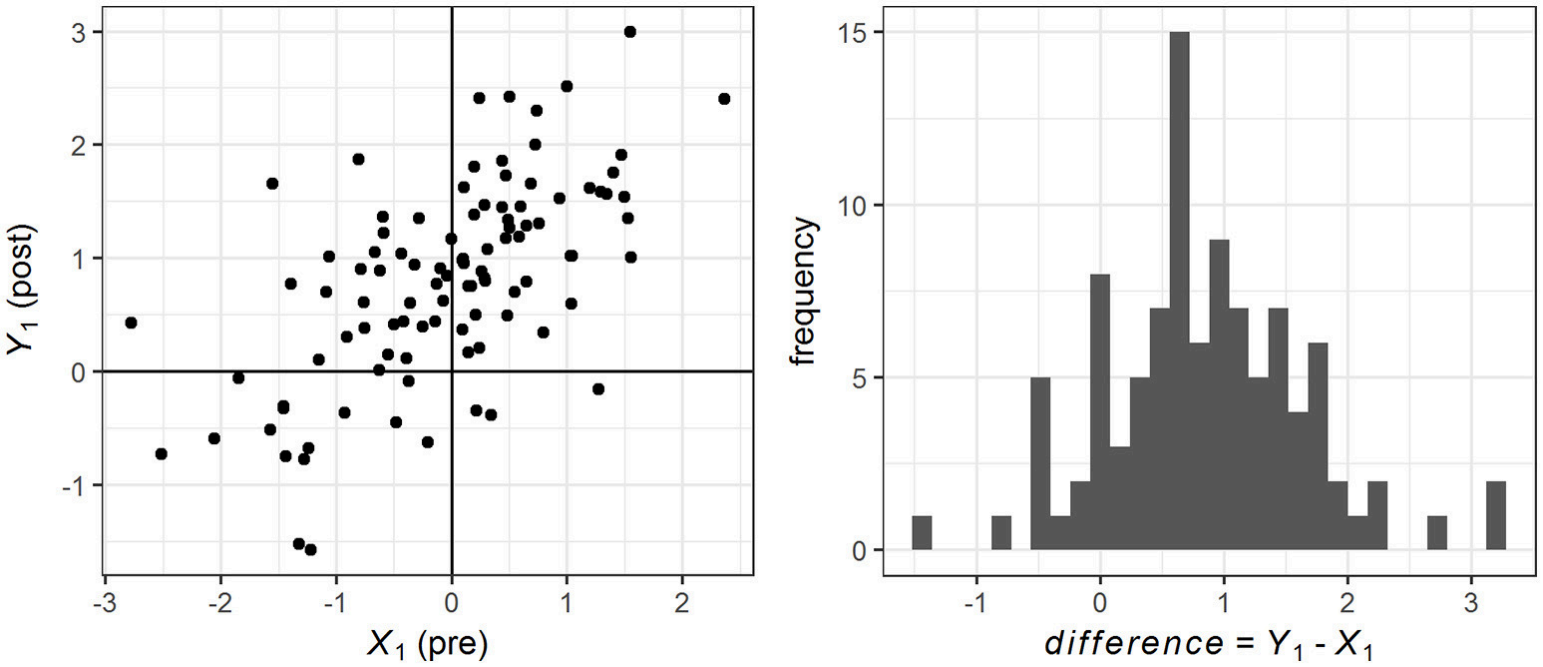
Pre, post and difference scores for one sample of *n* = 100, with δ_exp_ = 1.2, ρ_pre−post_ = 0.7, and normal distribution. Note that the amount of change is different for every individual.

To define the simulation conditions, we manipulated four criteria (for a summary, see Table 1):

a. *Effect size* in the experimental/treatment group (δ_exp_ = μ_dif.exp_/σ_dif.exp_). We computed the effect size as the standardized mean of the pre-post differences (Cohen, 1988; see the discussion and Appendix 1 in Supplementary Data Sheet 2 for considerations about using a different standardizer). We chose 13 effect sizes ranging from 0 to 3.6 with 0.3 point increases (e.g., an effect size of 0.6 indicates that the mean of the pre-post differences μ_dif.exp_ is 0.6 times the standard deviation of the individual pre-post differences σ_dif.exp_). The rationale for choosing this wide range of effects, from a null effect to an extremely large one, was to allow the percentage of individual changes to comprise its full range (0–100%). In our analyses we assumed that the mean scores increased over time. To calculate the differences, we subtracted pre-test score from the post-test score. Consequently, because we generated positive effects in our simulation, we used right one-tailed tests.
  In the single group pre-post design, we generated data for the treatment group only. In the control group pre-post design, we added data for a control group with no expected pre-post mean differences, (i.e., δ_ctrl_ = 0). The values for the rest of simulation criteria were the same for the control and treatment groups in every conditions (see below).
  Importantly, this value was the *mean* effect size in the population. Centered on this mean, a random distribution of individual changes was created, and each case within the sample experienced a different amount of change. The variance of this distribution depended on the pre-post correlation (see point *c* below). Figure 1 depicts the pre, post and change scores for one sample.
b. *Sample size of each group* (*n*). We chose three sample sizes (25, 50, and 100) to simulate what is usually considered small, medium, and large sample sizes in clinical work (Crawford and Howell, 1998). In the control group design, both groups had the same sample size.
c. *Pre-post correlation* (ρ_pre−post_): 0.5, 0.7, and 0.9. We chose these values to simulate a range of common correlations in applied settings (Pedhazur and Schmelkin, 1991; Nunnally and Bernstein, 1994. Note that correlations <0.5 are very uncommon in repeated measures settings). We used the Pearson's correlation coefficient. In the control group design, both groups were expected to have the same correlation value. With σ_pre_ = σ_post_ = 1, these three values lead to a standard deviation of the differences (σ_dif_) of 1, 0.775, and 0.447 respectively—i.e., higher pre-post correlation entails lower variance of the differences (See Appendix 3 in Supplementary Data Sheet 2 for a discussion on the effect of measurement error).
d. *Shape of the pre and post distributions*. Given that moderate and severe deviations from normality are often found in applied contexts (Micceri, 1989; Blanca et al., 2013), we simulated seven different conditions by modifying the degree of skewness (*g*_1_) and kurtosis (*g*_2_): (1) extreme negative skewness: *g*_1_ = −3, *g*_2_ = 18; (2) moderate negative skewness: *g*_1_ = −2, *g*_2_ = 9; (3) mild negative skewness: *g*_1_ = −1, *g*_2_ = 2; (4) normality: *g*_1_ = 0, *g*_2_ = 0; (5) mild positive skewness: *g*_1_ = 1, *g*_2_ = 2; (6) moderate positive skewness: *g*_1_ = 2, *g*_2_ = 9; and (7) extreme positive skewness: *g*_1_ = 3, *g*_2_ = 18. Note that the kurtosis is partially conditioned by the skewness. Less than 5% of real data is expected to have more extreme distributions (Blanca et al., 2013). In the control group design, both groups were expected to have the same shape for the pre- and post- distributions.

**Table 1:** Summary of simulation conditions and computed statistics.

	**Single group design**				**Control group design**			
**SIMULATION CONDITIONS**								
Effect size in the experimental group	δ_exp_ = μ_dif.exp_/σ_dif.exp_ = {0 to 3.6} in 0.3 steps				δ_exp_ = μ_dif.exp_ / σ_dif.exp_ = {0 to 3.6} in steps of 0.3 δ_ctr_ = μ_dif.ctr_ / σ_dif.ctr_ = 0			
Sample size	*n*_exp_ = {25, 50, 100}				*n*_exp_ = *n*_ctr_ = {25, 50, 100}			
Pre-post correlation	ρ_pre−post, exp_ = {0.5,0.7,0.9}				ρ_pre−post, exp_ = ρ_pre−post, ctr_ = {0.5,0.7,0.9}			
Shape of the pre and post distributions (equal for pre and post and for both groups)	Skew:	−3	−2	−1	0	1	2	3
	Kurt:	18	9	2	0	2	9	18
Average-based change statistic	*d* = (*M*_post_-*M*_pre_)/*S*_dif_				${\hat{\omega }}^{2}=\frac{gl_{AB}\left(F_{AB}-1\right)}{gl_{AB}\left(F_{AB}-1\right)+N}$			
Individual-based statistic (based on *SID* or *RCI*)	Percentage of reliable improvements				$P_{\text{net}}=\left(P_{\exp}^{+}-P_{\exp}^{-}\right)-\left(P_{\text{ctr}}^{+}-P_{\text{ctr}}^{-}\right)$			

### Simulation Procedure

By combining the four criteria described above we generated 13 × 3 × 3 × 7 = 819 different conditions for the simulation. For each of these conditions, we generated 500 samples (409,500 samples in total). This was done separately for the simple pre-post design (one experimental group per sample) and for the control group pre-post design (one experimental and one control group per sample). We used MatLab 2011a to perform the simulation. The code is available in the Supplementary Data Sheet 1.

In the single group design, we first generated a matrix **X**_**1**_ = (*X*_1_^*^, *Y*_1_^*^) containing *n* pairs of scores in two non-correlated variables. Scores were generated by using Pearson's distribution system. Both variables had the same mean, standard deviation, skewness, and kurtosis. The mean was always fixed to zero and the standard deviation was fixed to one. Skewness and kurtosis were systematically modified according to *g*_1_ and *g*_2_ values explained previously. *X* and *Y* were generated randomly to ensure that the post score may be the same, higher, much higher, lower or much lower than their corresponding pre-score, as is typically the case in real data.

Second, we fixed the correlation value between variables in **X**_**1**_ by applying the Cholesky covariance decomposition of correlation matrix **R** corresponding to the chosen correlation value (ρ_pre−post_). The resulting matrix **M**_**1**_ = (*X*_1_, *Y*_1_) contained two variables (*X*_1_ = pre; *Y*_1_ = post) with skewness, kurtosis and ρ_*XY*_ (or ρ_pre−post_) values similar to the specified ones. This transformation ensured that the post-scores were not independent of the pre-scores, as is also the case in real data. Note that, although simulating the difference scores would be simpler and faster than simulating pre and post scores, it would make it impossible to study the effect of the pre-post correlation.

In the last step we modified *Y*_1_ to adapt it to the desired mean value in each condition. For this purpose, we added the standard deviation of pre-post differences, multiplied by the corresponding value of δ_exp_, to each individual *Y*_1_ value.

In the control group design, the procedure was identical except for two changes: (a) instead of only one matrix in each replication, we generated a pair of independent matrices **X**_**1**_ = (*X*_1_^*^, *Y*_1_^*^) and **X**_**2**_ = (*X*_2_^*^, *Y*_2_^*^) for simulating the scores of the experimental (**X**_**1**_) and control (**X**_**2**_) groups; and (b) we modified *Y*_1_ in the experimental matrix only to adapt it to the desired mean value in each condition (whereas the mean for *Y*_2_ was not changed for the control group).

Importantly, this procedure ensured that every case experienced a different amount of change. Figure 1 depicts pre-, post- and difference scores for one sample of *n* = 100, with δ_exp_ = 1.2, ρ_pre−post_ = 0.7, and normal distribution.

### Data Analysis

In the single group pre-post design, we computed the empirical *group* or *average change* for each sample by calculating the difference between the post- and the pre-test means, and dividing such difference by the standard deviation of the differences,

(1) $$\begin{array}{l} \text{d}=\left({\text{M}}_{\text{post}}-{\text{M}}_{\text{pre}}\right)/{\text{S}}_{\text{dif}} \end{array}$$

In this paper we use *d* to refer to the result of applying Equation 1. See the discussion and Appendix 1 in Supplementary Data Sheet 2 for a discussion on a different computation of the standardized mean difference.

In the control group pre-post design, we quantified the average change by using the ω^2^ statistic associated with the interaction between the between-subjects factor *A* (group) and the within-subjects factor *B* (pre- and post-test). The net change is captured by comparing the pre-post change in the experimental group with the pre-post change in the control group (Hays, 1988; Kirk, 2013). For our design, ω^2^ can be estimated as

$$\begin{array}{l} \omega^{2}=\frac{gl_{AB}\left(F_{AB}\;-\;1\right)}{gl_{AB}\left(F_{AB}\;-\;1\right)\;+\;N}, \end{array}$$

were *F*_*AB*_ is the interaction *F* statistic, *gl*_*AB*_ are the interaction degrees of freedom, and *N* is the total number of scores in the design (adding both groups).

To identify which individual scores showed a reliable change (i.e., which cases fell above a certain cutoff after being standardized) and then calculate the percentage of individual changes for each sample, we decided to use two *individual change* indices. We chose two indices that have shown lowest false negative rates (see Ferrer and Pardo, 2014).

a. *Standardized individual difference* (*SID;* Payne and Jones, 1957). The standardized score resulting from dividing the individual pre-post difference (*D*_*i*_) by the standard deviation of these differences (*S*_dif_), as
  $$\begin{array}{l} SID=D_{i}/S_{\text{dif}}. \end{array}$$
  This standardization was proposed to assess the degree of discrepancy between two scores (Payne and Jones, 1957). If the distribution of pre-post difference is normal, 95% of *SID* will fall between ± 1.96 values, and 90% between ± 1.645 values.
b. *Reliable Change Index* (*RCI*; Jacobson et al., 1984, 1999; Jacobson and Truax, 1991). This is probably the most popular individual change index. It is based on the standard error of measurement. Of the several available versions, we used one in which the equality of pre- and post-test variances is not assumed (see Christensen and Mendoza, 1986; Jacobson and Truax, 1991; Maassen, 2004). This version is specified as:
  $$\begin{array}{l} RCI=\frac{D_{i}}{\sqrt{{\left(S_{\text{pre}}\sqrt{1-R_{\text{pre-post}}}\right)}^{2}+{\left(S_{\text{post}}\sqrt{1-R_{\text{pre-post}}}\right)}^{2}}}. \end{array}$$
  Using this index, the lower false positive rate is achieved when reliability is estimated from the pre-post correlation (*R*_pre−post_) (Ferrer and Pardo, 2014).

These two indices were computed for each individual case in all the simulated samples. We considered an individual change to be reliable when its corresponding *SID* or *RCI* was higher than 1.96 (two-tailed test) or 1.645 (one-tailed test) points. In the single group pre-post design, we applied one cutoff of 1.645. In the control group pre-post design we performed two-tailed tests (cutoffs of −1.96 and 1.96) for all conditions because the procedure is intended to compare the effectiveness of two different treatments in real scenarios. Hence, it is important to take into consideration the proportion of worsened cases, not only the improved ones.

In the single group design, we computed the percentage of reliable improvements for each sample. In the control group design, we computed the proportion of both worsened (*P*^−^) and improved (*P*^+^) cases in each group within the samples, and then subtracted the result for the control group (*ctrl*) from this same result in the experimental group (*exp*). This procedure yielded a net percentage of positive changes attributable to treatment^3^(*P*_net_):

(2) $$\begin{array}{l} P_{\text{net}}=\left(P_{\exp}^{+}-P_{\exp}^{-}\right)-\left(P_{\text{ctr}}^{+}-P_{\text{ctr}}^{-}\right) \end{array}$$

Then we examined the relation between the change estimated with ABC statistics and the change estimated using IBC statistics by fitting several regression functions.

Finally, with each empirical effect size and percentage of individual changes (500 pairs of values for each condition in the simulation, i.e., a pair by sample), we obtained: (a) a scatterplot to inspect the underlying relation between the two statistics, and (b) several different regression functions to quantify the extent to which the change in the distribution center is predictive of the percentage of individual changes. This was done separately for each research design.

## Results

For brevity of presentation, we report here the most representative results. For all conditions in both designs, the properties of the generated samples corresponded to those imposed in the simulation. We report the results regarding *SID* only; those based on the *RCI* are similar. Results from all conditions and based on the *RCI* are available upon request.

## Single Group Pre-Post Design

**Figure 2:**
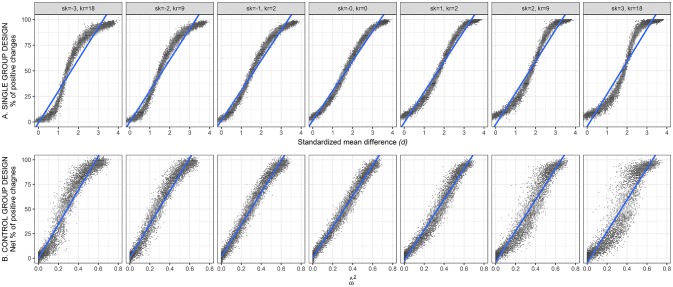
Relation between average-based change (horizontal axis) and individual-based change (vertical axis). Top row **(A)** shows the data for the simple group pre-post design. Bottom row **(B)** shows the data for the pre-post design with a control group. Data based on *SID* with *n* = 100 and ρ_pre−post_ = 0.7. Sk, skewness; Kr, Kurtosis.

To examine the relation between ABC and IBC, we first plotted the effect size measured by *d* (average-based change) against the percentage of individual changes (individual-based change). Figure 2 (top row) shows scatterplots based on *SID* index, for *n* = 100 and ρ_pre−post_ = 0.7. Each of the points in these scatterplot depicts one of the simulated samples (i.e., 13 effect sizes × 500 simulated samples = 6,500 points per scatterplot). The patterns with ρ_pre−post_ = 0.5 and ρ_pre−post_ = 0.9 were similar. We report here the conditions with the largest sample size to illustrate the shape of the relation with greater clarity. The same pattern is observed for *n* = 25 and *n* = 50, but with higher variability. In other words, any particular *d* value corresponds to the same mean percentage of changes, but a smaller sample size leads to more scattered points due to higher sampling error.

To quantify the relations detected in Figure 2, we estimated four different regression functions: linear, quadratic, cubic, and logistic. In every case, *d* (average-based effect size) was used as the independent variable and the percentage of changes (individual-based effect size) as the dependent variable.

Table 2 reports the coefficient of determination (*R*^2^) for the four functions, for *n* = 25. Because the dispersion in the various scatterplots decreases as sample size increases, these *R*^2^ values were the lowest of all values. Nevertheless, even with *n* = 25, three of the four functions provided an excellent fit. First, the linear function achieves *R*^2^ values around 0.90 in negatively skewed distributions and above 0.90 values in the remaining distributions, reaching 0.96. With *n* = 50 and *n* = 100, *R*^2^ ranges between 0.91 and 0.98; the lowest values are observed in the conditions with more extreme skewness. Second, the quadratic function achieves *R*^2^ values similar to the linear function, although slightly higher in negative skewness conditions. Third, the cubic function yields *R*^2^ values between 0.96 and 0.98, although at the cost of introducing more complexity. Fourth, the logistic function yields the lowest values, between 0.68 and 0.78.

**Table 2:** *R*^2^ of linear, quadratic, cubic and logistic functions for the single group design.

**Distribution**	**ρ_pre-post_**	**Linear**	**Quadratic**	**Cubic**	**Logistic**
Sk = −3, Kr = 18	0.5	0.919	0.938	0.965	0.695
	0.7	0.909	0.937	0.961	0.676
	0.9	0.897	0.940	0.956	0.681
Sk = −2, Kr = 9	0.5	0.937	0.951	0.974	0.701
	0.7	0.934	0.954	0.972	0.683
	0.9	0.928	0.955	0.971	0.679
Sk = −1, Kr = 2	0.5	0.951	0.960	0.979	0.705
	0.7	0.951	0.962	0.979	0.687
	0.9	0.946	0.962	0.977	0.679
Sk = 0, Kr = 0	0.5	0.962	0.965	0.982	0.723
	0.7	0.963	0.965	0.982	0.724
	0.9	0.962	0.964	0.982	0.722
Sk = 1, Kr = 2	0.5	0.956	0.956	0.979	0.740
	0.7	0.957	0.957	0.981	0.751
	0.9	0.955	0.956	0.980	0.756
Sk = 2, Kr = 9	0.5	0.943	0.943	0.975	0.752
	0.7	0.944	0.944	0.976	0.756
	0.9	0.939	0.942	0.973	0.770
Sk = 3, Kr = 18	0.5	0.927	0.927	0.969	0.757
	0.7	0.923	0.925	0.967	0.764
	0.9	0.915	0.921	0.960	0.779
Mean value		0.939	0.949	0.973	0.723
Min. value		0.897	0.921	0.956	0.676
Max. value		0.963	0.965	0.982	0.779

*n = 25; Independent variable: d; Dependent variable: percentage of changes based on SID; Sk, skewness; Kr, kurtosis*.

Three of the four adjusted functions offered a very good fit to the data. Moreover, they offered very similar predictions. For example, with *n* = 25, ρ_pre−post_ = 0.70, and δ_exp_ = 1, the predicted value (the estimated percentage of changes) is 30.7% for the linear function, 31.7% for the quadratic function, and 26.9% for the cubic function. Of these, the linear function is the most parsimonious, especially for applied settings (Bentler and Mooijaart, 1989; Maxwell and Delaney, 2004; Steele and Douglas, 2006). Table 3 reports the coefficients from the linear function. These coefficients can be used to estimate the percentage of individual changes from the effect size *d*. Given that the value of the former can range from −100 to 100, the constant coefficient *B*_0_ is fairly close to zero in every case (with absolute values ranging from 0.09 to 2.50, and standard errors <0.27; *p* > 0.05 in all cases), and the slope coefficient *B*_1_ is close to 30 (28.75 to 30.86, with standard error < 0.12). Results with other conditions were similar in all regards.

**Table 3:** Coefficients (and standard errors) for the lineal regression model in the single group design.

	**ρ_pre-post_** = **0.5**		**ρ_pre-post_** = **0.7**		**ρ_pre-post_** = **0.9**	
	***B*_**0**_**	***B*_**1**_**	***B*_**0**_**	***B*_**1**_**	***B*_**0**_**	***B*_**1**_**
Sk = −3, Kr = 18	−0.01 (0.24)	30.08 (0.11)	0.48 (0.25)	29.89 (0.12)	1.30 (0.27)	29.69 (0.12)
Sk = −2, Kr = 9	1.11 (0.20)	29.43 (0.09)	1.09 (0.21)	29.51 (0.10)	1.57 (0.22)	29.33 (0.10)
Sk = −1, Kr = 2	1.65 (0.18)	29.07 (0.08)	1.76 (0.18)	29.05 (0.08)	2.15 (0.18)	28.92 (0.09)
Sk = 0, Kr = 0	1.88 (0.15)	28.82 (0.07)	1.89 (0.15)	28.83 (0.07)	1.84 (0.15)	28.89 (0.07)
Sk = 1, Kr = 2	0.42 (0.17)	29.43 (0.08)	0.44 (0.16)	29.46 (0.08)	0.30 (0.17)	29.49 (0.08)
Sk = 2, Kr = 9	−0.49 (0.19)	29.93 (0.09)	−1.02 (0.19)	30.11 (0.09)	−1.12 (0.20)	30.12 (0.09)
Sk = 3, Kr = 18	−1.69 (0.22)	30.52 (0.11)	−2.45 (0.23)	30.78 (0.11)	−2.34 (0.24)	30.68 (0.12)

*n = 25; independent variable: d, dependent variable: percentage of changes based on SID. Sk, skewness; Kr, kurtosis*.

Results from the linear function indicate that: (a) when effect size is zero, the expected percentage of changes (computed using *SID*) ranges between 0 and 3%, and (b) for each extra point of effect size, the expected percentage of changes rises by 30 points. Because prediction is done using percentages, values below zero and above 100 must be replaced by their respective limits.

## Pre-Post Design With Control Group

Figure 2 (bottom row) shows the relation between ${\hat{\omega }}^{2}$ (average-based effect size measure) and the net percentage of changes (individual-based effect size measure). The latter was calculated from *SID* (*n* = 100 and ρ_pre−post_ = 0.7). Each of the points in these scatterplot depicts one of the simulated samples *comprising one control and one experimental group*. As in the top row, we report the results for the conditions with the largest sample size. The smaller sample sizes yielded the same pattern yet with higher variability. Patterns with the other ρ_pre−post_ values were similar.

To quantify the relation observed in the bottom row of Figure 2, we estimated four different regression functions: linear, quadratic, cubic and logistic. In every case, ${\hat{\omega }}^{2}$ was used separately as the independent variable, and the net percentage of individual changes served as the dependent variable. The four functions were estimated for each of the conditions simulated. Table 4 reports the coefficient of determination (*R*^2^) for these four functions. These results are based on net percentage of individual changes calculated with *SID* index and *n* = 25. Because dispersion from the various scatterplots decreases as sample size increases, *R*^2^ values from Table 4 were lower than those achieved with *n* = 50 and *n* = 100.

**Table 4:** *R*^2^ of linear, quadratic, cubic and logistic functions for the *n* = 25 conditions of the control group pre-post design.

**Distribution**	**ρ_pre-post_**	**Linear**	**Quadratic**	**Cubic**	**Logistic**
Sk = −3 Kr = 18	0.5	0.857	0.881	0.885	0.858
	0.7	0.857	0.887	0.890	0.858
	0.9	0.841	0.886	0.888	0.843
Sk = −2Kr = 9	0.5	0.894	0.907	0.911	0.894
	0.7	0.891	0.912	0.915	0.892
	0.9	0.887	0.917	0.920	0.888
Sk = −1Kr = 2	0.5	0.916	0.924	0.926	0.916
	0.7	0.916	0.927	0.929	0.916
	0.9	0.916	0.931	0.933	0.917
Sk = 0Kr = 0	0.5	0.926	0.929	0.930	0.926
	0.7	0.928	0.930	0.931	0.927
	0.9	0.924	0.926	0.928	0.924
Sk = 1Kr = 2	0.5	0.906	0.906	0.908	0.905
	0.7	0.902	0.902	0.903	0.901
	0.9	0.898	0.898	0.900	0.898
Sk = 2Kr = 9	0.5	0.866	0.867	0.868	0.867
	0.7	0.865	0.865	0.867	0.865
	0.9	0.841	0.841	0.843	0.842
Sk = 3Kr = 18	0.5	0.824	0.825	0.827	0.825
	0.7	0.815	0.816	0.817	0.816
	0.9	0.785	0.786	0.787	0.785
Mean value		0.879	0.889	0.891	0.879
Min. value		0.785	0.786	0.787	0.785
Max. value		0.928	0.931	0.933	0.917

*Independent variable: ${\hat{\omega }}^{2}$. Dependent variable: net percentage of individual changes based on SID. Sk, skewness; kr, Kurtosis*.

Overall, the four functions achieved a very good fit. The *R*^2^ values were higher when the distributions approached normality. The quadratic and cubic functions achieved a slightly better fit than the linear function, but only with negative skewness; the logistic and linear functions achieved similar fit. As in the single group design, the linear function was deemed preferable because it is the most parsimonious, with only minimal loss of fit.

In Table 5 (analogous to Table 3 in the single group design) we report the coefficients from the linear function with *n* = 25. These coefficients allow estimating the net percentage of individual changes from the effect size measures. The intercept (*B*_0_) ranges from −0.04 to approximately 6, with a mean of 2.41 and standard errors ranging between 0.19 and 0.33. The slope (*B*_1_) ranges from 140 to 165, with a mean of 153 and standard errors ranging between 0.53 and 0.91. As an example, if we consider the results for the normal distributions, these coefficients indicate that for a null effect size (${\hat{\omega }}^{2}$ = 0), the linear function yields an estimated net percentage of changes of approximately 2.5%. For each additional 0.10 points of ${\hat{\omega }}^{2}$, the net percentage of changes increases in approximately 15 points (as we are predicting percentages, values beyond zero, and 100 must be replaced by their respective limits). Note that the changes in pre-post correlation do not substantially alter the coefficients *B*_0_ and *B*_1_ in Table 5. Similar results were found with the other sample sizes.

**Table 5:** Coefficients (and standard errors) for the lineal regression model in the design with a control group.

	**ρ_pre-post_** = **0.5**		**ρ_pre-post_** = **0.7**		**ρ_pre-post_** = **0.9**	
	***B*_**0**_**	***B*_**1**_**	***B*_**0**_**	***B*_**1**_**	***B*_**0**_**	***B*_**1**_**
Sk = −3, Kr = 18	3.51 (0.29)	162.9 (0.82)	4.18 (0.29)	164.7 (0.83)	5.92 (0.31)	164.9 (0.89)
Sk = −2, Kr = 9	3.02 (0.24)	160.3 (0.69)	3.95 (0.25)	162.0 (0.70)	5.18 (0.25)	162.5 (0.72)
Sk = −1, Kr = 2	3.49 (0.21)	156.7 (0.59)	3.74 (0.21)	157.5 (0.59)	4.50 (0.21)	158.2 (0.59)
Sk = 0, Kr = 0	2.35 (0.19)	152.2 (0.53)	2.56 (0.19)	151.7 (0.53)	2.54 (0.19)	151.8 (0.54)
Sk = 1, Kr = 2	1.07 (0.21)	149.8 (0.60)	0.86 (0.22)	147.6 (0.61)	0.37 (0.22)	146.6 (0.61)
Sk = 2, Kr = 9	0.96 (0.26)	148.8 (0.73)	0.33 (0.26)	146.2 (0.72)	−0.03 (0.28)	143.4 (0.77)
Sk = 3, Kr = 18	0.95 (0.30)	147.6 (0.84)	0.63 (0.31)	144.1 (0.85)	0.43 (0.33)	139.7 (0.91)

*n = 25; independent variable: ${\hat{\omega }}^{2}$; dependent variable: net percentage of individual changes based on SID. Sk, skewness; Kr, kurtosis*.

## Discussion

Our first goal in this paper was to determine whether ABC (quantified by *d* in the single group design or by ${\hat{\omega }}^{2}$ in the control group design) is related to IBC (quantified as the percentage of individual changes, or net percentage in the control group design). Our simulations indicate that percentage of changes is related to average-based effect size. In all conditions, and for both designs, the results show that, as average-based effect size increases, so does the percentage of changes.

Within this general goal, we aimed at finding a mathematical function to capture the relation between effect size and percentage of changes. In both designs, the adjusted linear, quadratic and cubic functions showed excellent fit. The logistic function showed good fit in the single group design, and excellent fit in the control group design. Among them, the linear model was the most parsimonious and easiest to interpret, and hence was preferred (Bentler and Mooijaart, 1989; Maxwell and Delaney, 2004; Steele and Douglas, 2006). It showed excellent fit in all conditions even in the least favorable simulated scenarios (*n* = 25): the *R*^2^ values ranged from 0.90 to 0.96 in the single group design (Table 2) and from 0.79 to 0.93 in the control group design (Table 4).

Finally, we wanted to identify conditions in which the ABC and IBC are related. Our results indicate that such a relation was present in all simulated conditions and for both designs, regardless of the pre and post distributions skewness, and of the pre-post correlation. The fit (*R*^2^) of the linear regression function slightly varied from 0.96, in the most favorable conditions, to 0.90 (single group design), and 0.79 (control group design) in the most adverse. As sample size increases, so does fit: with *n* = 100, *R*^2^ reached 0.98 in the most favorable conditions, and was never below 0.87 in the most adverse.

A very important finding from our study was that, for both designs, *the slope of the regression line was approximately the same in all simulated conditions*. In the single group design (with *d* as predictor and the percentage of changes as dependent variable), the slope value was around 30 (ranging from 29 to 31). This indicates that, for each added point to the effect size, the function's estimation of the percentage of changes increased by 30 points. In other words, a 0.10-point increase in *d* (pre-post differences metric) was associated with a 3-point increase in the percentage of individual changes^4^.

In the control group design (with ${\hat{\omega }}^{2}$ as predictor and the *net* percentage of changes as the dependent variable), the slope value was around 153 points, ranging from 140 to 165. Because the values of ${\hat{\omega }}^{2}$ range from 0 to 1, expressing it this way is more useful: for each 0.10 added points to the effect size, the linear function estimate for the percentage of individual changes increases in 15.3 points (ranging from 14 to 16.5).

### Relevance of the Present Findings

Some important implications are worth noting: (a) The ABC and IBC statistics are nearly equivalent; and (b) Cutoffs commonly used for deciding when an effect is small, medium or large should be replaced with more informative indices. Below, we expand on these ideas and offer two recommendations based on them.

#### The ABC and IBC Statistics Are Nearly Equivalent

With two exceptions (Norman et al., 2001; Lemieux et al., 2007), papers on this topic agree on the following idea: researchers will arrive to different conclusions about a treatment's effectiveness depending on whether they assess it at the individual or at the group level (e.g., Ottenbacher et al., 1988; Testa, 2000; Schmitt and Di Fabio, 2004; Vindras et al., 2012). Our results indicate that this idea is incorrect. Across all of our simulation conditions, ABC and IBC statistics were so closely related that can be considered as different expressions of nearly the same information. This is to be expected, indeed, when variability of pre- and post-test scores is the same. Because increases in effect size lead to increases in the center of the pre-post differences distribution, the number of cases on the right side of any chosen cutoff will also increase.

Based on this finding, we offer our first **recommendation:** When evaluating the change in a group, if only one pre- and one post- measures are available, a logical sequence of analytic steps is as following: (a) assess individual changes through *SID* or *RCI*, (b) aggregate the individual results into a percentage of reliable individual changes (or net percentage, if more than one group is analyzed), and, (c) report this individual-based statistics *along with* classical average-based effect size estimations such as *d* or ${\hat{\omega }}^{2}$.

This procedure has several advantages over just reporting the ABC statistics. First, it allows researchers to make decisions about each particular case. This is a common concern in applied settings, and the individual-based methods discussed here provide a straightforward tool for addressing it (Sijtsma, 2012). The usefulness and convenience of these indices have been discussed elsewhere (Jacobson and Truax, 1991; Maassen, 2000; Ferrer and Pardo, 2014). Second, an effect size expressed as a percentage is easier to understand and it enhances the communication of results, especially among researchers without a strong statistical background. For example, in a randomized controlled trial, stating that the effect size was ${\hat{\omega }}^{2}$ = 0.20 is less clear than stating that the observed net percentage of individual changes was 33%.

Recent recommendations advocate that effect size estimates should directly address the research question which motivated their estimation, and should be intuitively accessible so that they facilitate the constructive scrutiny of results (Pek and Flora, 2018). We argue that, when used for effect size interpretation, individual-based statistics accomplish both aims. Based on this, and in line with previous work (e.g., Ogles et al., 2001; Wise, 2004; Lambert and Ogles, 2009; Speelman and McGann, 2013; de Beurs et al., 2016; Fisher et al., 2018), we encourage other researchers to include individual-based statistics in their methodological toolbox and to use them to report their results.

Another finding worth highlighting is that, because the intercept and slope coefficients were very similar across conditions, it is easy to compute an approximate percentage (or net percentage) of reliable individual changes *even without having access to the raw data*. For example, if a researcher wants to express an already published effect size as a percentage of changes, the only needed step is to introduce the estimate into the linear regression equation proposed in our results. For example, in a single group pre-post study with *d* = 0.9 with normally distributed scores, and based on Table 3:

(3) $$\begin{array}{l} Percentage\;of\;changes\approx B_{0}+B_{1}\times d=1.9+29\times 0.9\approx 28\% \end{array}$$

In a control group pre-post study with ${\hat{\omega }}^{2}$ = 0.4, with normally distributed scores, pre-post correlation of *r* = 0.7, and based on Table 5:

(4) $$\begin{array}{l} Net\;percentage\;of\;changes\approx {\text{B}}_{0}+{\text{B}}_{1}\times {\hat{\omega }}^{2}=2.6+152\times 0.4\approx 63\% \end{array}$$

When *d* or ${\hat{\omega }}^{2}$ are not available in the published report, it is easy to compute them from other effect sizes estimates (see Appendix 1 in Supplementary Data Sheet 2 for examples of these computations, and see Appendix 2 in Supplementary Data Sheet 2 for an application to data from one published paper). The specific intercept and slope values can be selected according to the empirical skewness and kurtosis (see Tables 3, 5). But even if coefficients from a wrong condition are selected, the estimate of the (net) percentage of changes will be close to the real value.

Based on previous research (Blanca et al., 2013), less than 5% of real datasets have more extreme distributions than the ones simulated here. Consequently, our simple linear regression models can be applied in most real situations to estimate the approximate percentage of individuals who experienced change, when only average-based change indicators are available. Of course, when possible, computing the actual empirical value is preferable.

#### Cutoffs Commonly Used for Deciding When an Effect Is Small, Medium or Large Should be Replaced With More Informative Indices

In many contexts, it is frequent to use cutoffs to interpret the magnitude of an effect. The cutoffs proposed by Cohen (1988) are arguably the most popular. When considering these cutoffs for identifying small, medium and large effect sizes, we find that, in our simulated single group pre-post scenarios, a small effect (*d* = 0.2) corresponds to 8% of changes, a medium effect (*d* = 0.5) corresponds to 17%, and a large effect (*d* = 0.8) corresponds to 26%. Similar guidelines have been proposed for control group pre-post designs (e.g., Kirk, 2013). According to our results, the proposed ${\hat{\omega }}^{2}$ values for declaring a small, moderate, and large effect size (0.01, 0.06, and 0.14) would lead to 4, 12, and 24% of net percentage of changes, respectively. In both designs, the idea that a so-called large effect size leads to just 24–26% of changes (or net changes) does not seem reasonable.

Based on our findings, we recommend that arbitrary cutoffs for evaluating the magnitude of effect estimates should not be used. We are not proposing a new set of cutoffs; rather, we propose to stop using them altogether. Indeed, other authors have suggested this idea before (e.g., Hill et al., 2008; Pek and Flora, 2018), but researchers still use arbitrary guidelines and cutoffs because they are useful for making sense of their findings. Particularly in clinical, educational, and other substantive domains, applied practitioners need to *know the meaning* of values such as *d* = 0.6, *r* = 0.4, ${\hat{\eta }}^{2}$ = 0.4, or ${\hat{\omega }}^{2}$ = 0.35. Arbitrary cutoffs are appealing as easy rules of thumb, despite their many disadvantages.

**Our second recommendation** is to use individual-based statistics as a simple tool for interpreting the magnitude of empirical effects. We illustrate this idea with a simple example. Suppose a researcher wants to assess the effectiveness of a new treatment for the pathological fear of darkness. A sample of 100 patients with this fear is gathered and randomly assigned to two groups (treatment group, receiving the new intervention, and control group, receiving no intervention). After finishing the program, the researcher obtains an average-based effect size of ${\hat{\omega }}^{2}$ = 0.26 for the interaction between group and occasion of measurement. Instead of declaring that the effect is “large” (Kirk, 2013), the researcher also computes a net percentage of changes (based on Table 5),

$$\begin{array}{l} Net\;percentage\;of\;changes\approx B_{0}+B_{1}\times {\hat{\omega }}^{2}=2.6+152\times 0.26\approx 42\%. \end{array}$$

Using the individual-based statistic and substantive knowledge on the disorder, he decides to discard the new intervention in favor of the traditional one, because they usually achieve much higher rates of success. Now, suppose that a different researcher wants to assess the effectiveness of a new treatment for autism in 10- year old children. She applies the new intervention using the exact same sample size and research design, and finds the same effect sizes estimates. In the context of an intervention to treat autism spectrum disorders, she can arguably claim that the effect is “very large” (indeed, she can claim the Nobel Prize).

In both cases, the researchers can easily decide whether ${\hat{\omega }}^{2}$ = 0.26 means a “small” or a “large” effect based on: (a) the individual-based statistic; and (b) their theoretical knowledge on the substantive domain. The individual-based statistics help interpreting the meaning of the effect size estimation but, unlike arbitrary “general guidelines,” do not force researchers to interpret them invariantly across different domains. By using them, applied practitioners can easily understand and communicate the meaning of any value of the percentage of changes in the context of their particular field.

### Theoretical and Methodological Considerations, and Future Directions

In our analyses we used the standard deviation of the pre-post differences (σ_dif_) as the standardizer of our single-group ABC statistic, but other standardizers are also available. For example, one common procedure is to use the standard deviation of the pre- scores (σ_pre_). The choice of the standardizer is related to the ability of the effect size measure to deal with pre-post dependency. Using σ_dif_ allows taking into account such dependency because σ_dif_ is partially dependent on the pre-post correlation, but there is no consensus on the correct procedure, and different authors advocate for different solutions (Gibbons et al., 1993; Dunlap et al., 1996; Morris and DeShon, 2002; Ahn et al., 2012).

A full discussion of the implications of using different standardizers is beyond the scope of this study, and we refer the reader to the aforementioned literature. However, it is important to note that using σ_pre_ as the standardizer for *d* will affect the relation between the ABC and IBC statistics. Specifically, the *B*_1_ coefficient in Equation 3, which captures the regression slope, will have higher values for higher levels of pre-post correlation. In other words, although the relation can be considered linear regardless of the standardizer chosen, the slope of such linear function will differ depending on the pre-post correlation if σ_pre_ is used. In contrast, it will remain constant if σ_dif_ is chosen. See Appendix 1 in Supplementary Data Sheet 2 for a more detailed description and some examples.

In a different vein, some caution is warranted when interpreting IBC statistics. For example, suppose that a researcher assesses the change in academic achievement from a given grade to the next in a single school group, and she finds an effect size of *d* = 0.3. With normally distributed scores, and according to our Equation 3,

$$\begin{array}{l} Percentage\;of\;changes\approx B_{0}+B_{1}\times d=1.9+29\times 0.3\approx 11\% \end{array}$$

This value does not imply that 89% of the students did not learn. Instead, it indicates that, given the observed variability in the pre-post differences, only 11% of such improvements could be identified as reliable. The same mean difference (say, for example, 10 IQ points) combined with a lower value of σ_dif_ would lead to a higher value of both *d* and the percentage of changes. Note that this “attenuation problem” affects both the ABC and IBC statistics. Other factors such as measurement error also attenuate the value of both types of statistics (see the Appendix 3 in Supplementary Data Sheet 2). However, IBC statistics should always be interpreted in the context of a particular research domain, and it is reasonable to think that measurement error, “natural” variability in the differences (σ_dif_), and other attenuating factors, will remain fairly constant across studies from the same domain—particularly if they use the same measurement instrument. If more than two measurement occasions are available, other statistical tools can be used to assess individual change (e.g., Estrada et al., 2018). These tools are particularly useful for examining developmental and learning processes, and can incorporate measurement error.

In our simulated scenarios, both groups were expected to have scores with the same distributional shape and dispersion in the pre- and post- evaluations—i.e., only the center of the distribution was expected to change. Of course, the distribution shape and variability can also change between both assessments, for example, as a result of an intervention. It is unclear whether our findings apply to such scenarios, and future research should address this important point.

## Conclusion

In this paper we show that individual- and average-based statistics for measuring change are closely related, regardless of sample size, pre-post correlation, and shape of the scores' distribution. To our knowledge, this is the first study applying individual reliable change indices to an experimental design. Our findings are relevant for a range of scientific disciplines including education, psychology, medical and physical therapy. We encourage other researchers to use individual change indices and individual-based statistics. Their main advantages are: (a) they allow determining which individual cases changed reliably; (b) they facilitate the interpretation and communication of results; and (c) they provide a straightforward evaluation of the magnitude of empirical effects while avoiding the problems of arbitrary general cutoffs.

## Author Contributions

AP developed the original idea. EE and AP reviewed the relevant literature. EE and AP designed the study and data analysis strategy. EE conducted the data simulation. EE, EF, and AP analyzed the data and interpreted the results, organized the article structure and drafted the manuscript critically revised the manuscript.

### Conflict of Interest Statement

The authors declare that the research was conducted in the absence of any commercial or financial relationships that could be construed as a potential conflict of interest.

## Footnotes

^1^In some cases, interventions aim at decelerating or stopping changes that are already happening: i.e., an intervention for the elderly aiming at stopping or reducing the speed of decline of some cognitive function. In such situations, “no change” is evidence of treatment success.

^2^In computing the *distribution center* all cases are used, each one of them contributing its proportional share of change; in computing the *percentage of changes* only cases above a given cutoff are involved and, moreover, all of them equally weighted regardless of their change.

^3^Norman et al. (2001), inspired on Guyatt et al. (1998), proposed an alternative *corrected* method for computing *P*_net_:

$$\begin{array}{l} P_{\text{net}}=\left(P_{\exp}^{+}-P_{\exp}^{-}\right)-\left(P_{\text{ctrl}}^{+}-P_{\text{ctrl}}^{-}\right)-P_{\exp}^{-}\times P_{\text{ctrl}}^{+}-P_{\exp}^{+}\times P_{\text{ctrl}}^{-} \end{array}$$

Since the results obtained with this equation and with [2] and are nearly identical, here we will only inform about the results obtained with [2].

^4^For a correct interpretation of these results, it should be noted that if the *Y* variable ranges from zero to 100 (as percentage of changes does) and the *X*-*Y* relation is perfect, the *Y* slope value equals 100 divided by the *X* range. In our case, if the relationship between *X* (effect size) and *Y* (percentage of changes) were perfect, the slope of the regression line will be equal to 100/3.6 = 27.8. The slopes found in this study ranged from 29 to 31 because the studied relationships were not perfect. This only means that, in order to find the correct slope, it is important to take into consideration a range of *X* values which allows working with all possible values of *Y*. Our results show that the chosen range of effect size values allowed us to study the complete range of percentages of individual changes

## Supplementary Material

Supplementary Data Sheet 1 Simulation code.

Supplementary Data Sheet 2 Appendices.
