# Supplementary material for: Statistics for Evaluating Pre-post Change: Relation Between Change in the Distribution Center and Change in the Individual Scores
Source: Front Psychol. 2019 Jan 8;9:2696. doi: 10.3389/fpsyg.2018.02696 (PMC6331475; doi:10.3389/fpsyg.2018.02696)
Supplement: Supplementary Data Sheet 2 — Appendices. [file Data_Sheet_2.pdf]

# APPENDIX 1

## Alternative average-based effect sizes and their relation to individual-based statistics

### *A1.1. Single group pre-post design: Computation of Cohen's d using the standard deviation of the pre scores as standardizer*

For a pre-post design, effect size is usually estimated as the standardized mean of the pre-post differences (Cohen, 1988) but standardization can be computed in two different ways: a) the mean of the pre-post differences ($\mu_{dif}$) is divided by the standard deviation of pre scores ($\sigma_{pre}$), or b) it is divided by the standard deviation of pre-post differences ($\sigma_{dif}$).

Sometimes, researchers prefer to use $\sigma_{pre}$ because such variability of the pre scores is a natural reference for thinking about original scores, as opposed to the variability of the differences ($\sigma_{dif}$) (Cohen, 1988; Cumming & Finch, 2001). Importantly, using a different standardizer for δ does not alter the findings in this study: the strong relation between ABC and IBC holds, regardless of whether we use $\delta_1 = \mu_{dif} / \sigma_{dif}$ or $\delta_2 = \mu_{dif} / \sigma_{pre}$ as the ABC.

The main consequence of using $\delta_2$ is the fact that it leads to different slope coefficients ($B_1$) for different values of pre-post correlation –or standard deviation of the differences. In other words, the regression equation describing the relation –and its predictions– vary as a function of the pre-post correlation. This is represented in Figure A.1, which depicts samples of size 100 with normally distributed scores and different values of pre-post correlation.

**Figure A.1.** Relation between average-based change (horizontal axis) and individual-based change (vertical axis), as a function of the standardizer used for computing Cohen's δ. Note: Data based on *SID* with *n* = 100 and normal distribution.

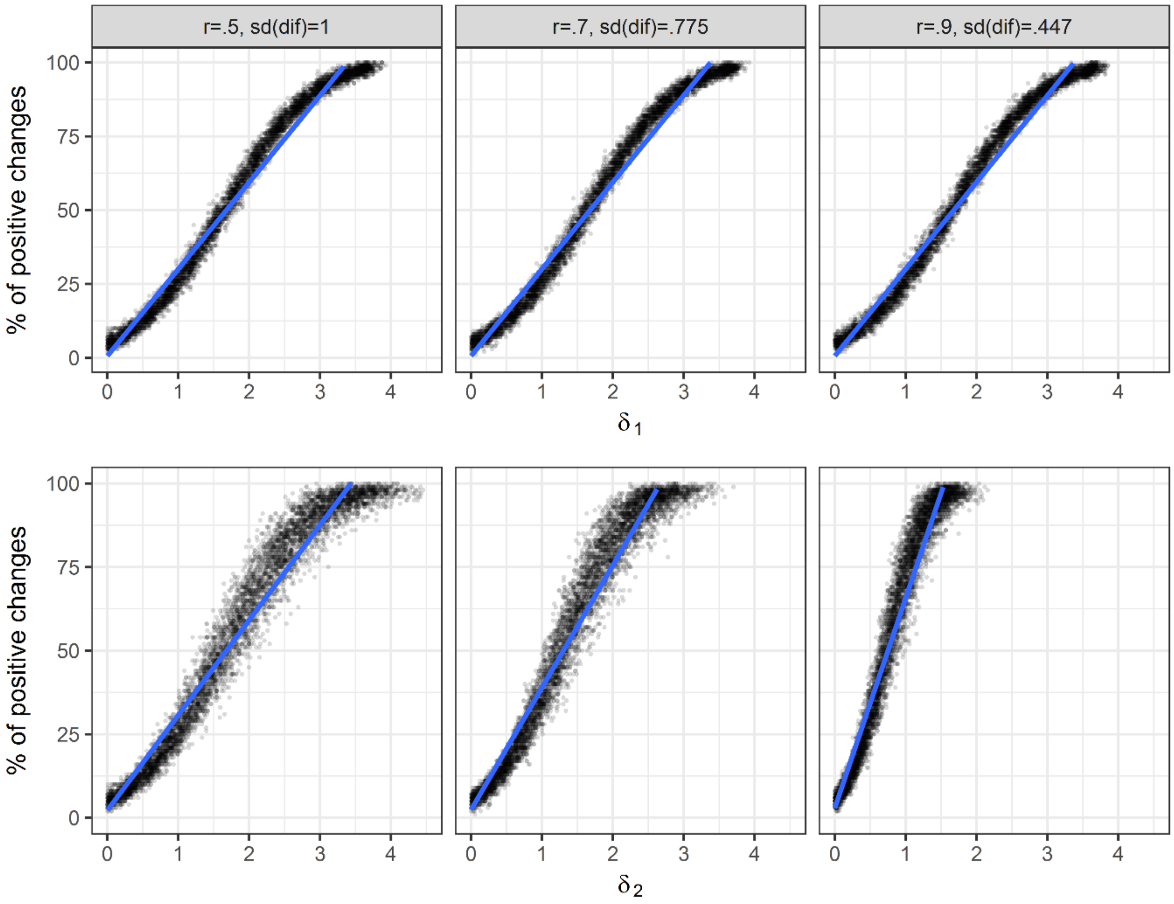


When the percentage of positive changes is regressed on $\delta_2$ (bottom panel in Figure A.1), values of pre-post correlations of .5, .7 and .9 lead to $B_1$ values of .281, .363 and .624, respectively. The adjusted $R^2$ in these three cases were .951, .948 and .943. This suggests that the relation between ABC and IBC does not change depending on the standardizer, besides the expected change in the metrics captured in the $B_1$ coefficient. If the researcher needs of convert one form into the other, the relation between both of them is given by (Cohen, 1988): $\delta_1 = \delta_2\,[2\,(1 - \rho_{\text{pre-post}})]^{1/2}$

*<u>A1.2. Pre-post design with a control group: Using a "d-type" effect size estimate</u>*

For this design, we have used $\omega^2$ as our effect size estimate. This is an "*r*-type" statistic based on the percentage of variance explained by the interaction effect between group and time. However, a pre-post design with a control group also allows the computation of "*d*-type" effect size measures, based on the standardization of the mean differences. Although *r*-type and *d*-type effect estimations are often interchangeable, *d*-type statistics are more frequent in some research fields.

In a control group pre-post design, the treatment effect can be estimated by a version of Cohen's standardized difference (1988; Grissom & Kim, 2012; Morris, 2008) for interaction effect in pre-post designs with control group (*PPC*):

$$d_{PPC} = \frac{\left(M_{\text{post;exp}} - M_{\text{post;ctrl}}\right) - \left(M_{\text{pre;exp}} - M_{\text{pre;ctrl}}\right)}{S_{\text{pre}}} \quad \text{[A.1]}$$

where $M$ is the mean and $S_{\text{pre}}$ is the pooled standard deviation in the pre-test, which can be calculated (with $n_{\text{exp}} = n_{\text{ctrl}}$) as $S_{\text{pre}} = \sqrt{\left(S^2_{\text{pre;exp}} + S^2_{\text{pre;ctrl}}\right)/2}$ .

In Table A.1 we report the coefficient of determination ($R^2$) for the linear, quadratic, cubic and logistic functions when $d_{\text{PPC}}$ is used as the independent variable and the net percentage of individual changes as the dependent variable, calculated with *SID* index and $n = 25$. As with $\hat{\omega}^2$, the four functions achieve a very good fit.

**Table A.1**

$R^2$ of linear, quadratic, cubic and logistic functions for the $n$ = 25 conditions of the control group pre-post design.

| | | $d_{PPC}$ as independent variable | | | |
|---|---|---|---|---|---|
| Distribution | $\rho_{pre\text{-}post}$ | Linear | Quad. | Cubic | Log. |
| Sk = -3 Kr = 18 | .5 | .816 | .850 | .851 | .817 |
| | .7 | .834 | .883 | .883 | .836 |
| | .9 | .829 | .892 | .892 | .832 |
| Sk = -2 Kr = 9 | .5 | .862 | .882 | .883 | .862 |
| | .7 | .873 | .902 | .903 | .873 |
| | .9 | .879 | .918 | .920 | .880 |
| Sk = -1 Kr = 2 | .5 | .891 | .902 | .903 | .891 |
| | .7 | .900 | .915 | .916 | .900 |
| | .9 | .908 | .926 | .928 | .908 |
| Sk = 0 Kr = 0 | .5 | .910 | .913 | .914 | .909 |
| | .7 | .914 | .917 | .918 | .913 |
| | .9 | .919 | .923 | .924 | .919 |
| Sk = 1 Kr = 2 | .5 | .893 | .894 | .896 | .893 |
| | .7 | .898 | .898 | .900 | .898 |
| | .9 | .896 | .896 | .898 | .896 |
| Sk = 2 Kr = 9 | .5 | .864 | .867 | .869 | .864 |
| | .7 | .868 | .869 | .872 | .868 |
| | .9 | .848 | .848 | .850 | .848 |
| Sk = 3 Kr = 18 | .5 | .826 | .829 | .832 | .826 |
| | .7 | .826 | .827 | .829 | .826 |
| | .9 | .795 | .795 | .797 | .796 |
| Mean value | | .869 | .883 | .885 | .869 |
| Min. value | | .795 | .795 | .797 | .796 |
| Max. value | | .919 | .923 | .924 | .919 |

Note: Independent variable: $d_{PPC}$. Dependent variable: net percentage of individual changes based on *SID*. Sk = skewness; Kr = Kurtosis.

In Table A.2 we report the coefficients from linear function with $n = 25$. In contrast to $\hat{\omega}^2$, the coefficients for $d_{PPC}$ are strongly affected by the pre-post correlation: they increase with higher values of $\rho_{pre\text{-}post}$ (the effect is particularly clear for the slope $B_1$). This is expected if one considers that $d_{PPC}$ is based on the standard deviations of pre scores, while the net percentage of individual changes is obtained applying change indices (*SID*, *RCI*) based on the standard deviation of pre-post differences. The former does not incorporate the pre-post relationship, while the later does.

**Table A.2**

Coefficients (and standard errors) for the lineal regression model in the design with a control group

| | | $\rho_{pre\text{-}post} = .5$ | | $\rho_{pre\text{-}post} = .7$ | | $\rho_{pre\text{-}post} = .9$ | |
|---|---|---|---|---|---|---|---|
| | | $B_0$ | $B_1$ | $B_0$ | $B_1$ | $B_0$ | $B_1$ |
| $d_{PPC}$ | Sk = -3, Kr = 18 | 8.05 (.46) | 21.91 (.20) | 10.48 (.48) | 27.27 (.26) | 15.19 (.52) | 43.98 (.49) |
| | Sk = -2, Kr = 9 | 3.47 (.39) | 24.29 (.17) | 6.17 (.41) | 30.42 (.23) | 8.51 (.43) | 51.35 (.42) |
| | Sk = -1, Kr = 2 | .04 (.32) | 26.46 (.14) | 1.18 (.33) | 33.81 (.20) | 3.12 (.35) | 56.61 (.35) |
| | Sk = 0, Kr = 0 | -1.64 (.28) | 26.38 (.13) | -1.41 (.28) | 33.68 (.17) | -1.38 (.29) | 58.16 (.29) |
| | Sk = 1, Kr = 2 | -1.39 (.32) | 24.79 (.15) | -1.32 (.33) | 31.32 (.19) | -1.18 (.33) | 52.95 (.33) |
| | Sk = 2, Kr = 9 | 2.34 (.39) | 22.00 (.17) | 2.46 (.39) | 27.48 (.22) | 3.19 (.40) | 45.02 (.39) |
| | Sk = 3, Kr = 18 | 6.37 (.45) | 19.14 (.19) | 6.66 (.45) | 23.66 (.24) | 6.84 (.45) | 38.61 (.41) |

Note: $n = 25$; independent variable: $d_{PPC}$; dependent variable: net percentage of individual changes based on *SID*). Sk = skewness; Kr = kurtosis.

To illustrate this idea, in Table A.3 we report the correspondence between $d_{PPC}$ and the net percentage of individual changes for some values of $\rho_{pre\text{-}post}$. Therefore, when using $d_{PPC}$ for estimating the net percentage of changes, it is necessary to correct the estimation incorporating the relationship between pre and post scores. One way to do this is by using $\hat{\omega}^2$ to compute the estimates, after transforming $d_{PPC}$ into $\hat{\omega}^2$ by:

$$\omega^2 = \frac{d_{\text{PPC}}^2\ n_{\text{exp}}\ n_{\text{ctrl}}\ S_{\text{pre}}^2 - \left(n_{\text{exp}} + n_{\text{ctrl}}\right) S_{\text{dif}}^2}{d_{\text{PPC}}^2\ n_{\text{exp}}\ n_{\text{ctrl}}\ S_{\text{pre}}^2 + \left(n_{\text{exp}} + n_{\text{ctrl}}\right)\left(4n_{\text{exp}} - 1\right) S_{\text{dif}}^2}\ . \qquad \text{[A.2]}$$

**Table A.3**

Relationship between $d_{\text{PPC}}$ and net benefit based on *SID* ($n$ = 25 and normal distribution).

| | $\rho_{\text{pre-post}}$ = .5 | $\rho_{\text{pre-post}}$ = .7 | $\rho_{\text{pre-post}}$ = .9 |
|---|---|---|---|
| $d_{\text{PPC}}$ = .2 implies a net % of changes of… | 3.6% | 5.3% | 10.3% |
| $d_{\text{PPC}}$ = .5 implies a net % of changes of… | 11.6% | 15.4% | 27.7% |
| $d_{\text{PPC}}$ = .8 implies a net % of changes of… | 19.5% | 25.5% | 45.1% |
| net 100% of changes is reached with $d_{\text{PPC}}$ = | 3.73 | 2.93 | 1.70 |

## APPENDIX 2

### Computation of the net percentage of changes using data from a published study

Lowrie, Logan & Ramful (2017) applied a 10 week visual-spatial intervention program for improving spatial reasoning and mathematics performance in 120 students of ages 10-12. They compared the pre- and post- scores with those of 66 control students. According to their Table 3, in the intervention group, the $t_1$ and $t_2$ means of the spatial visualization task were 6.28 (*Sd* = 2.55) and 7.52 (*Sd* = 2.75). In the control group, the $t_1$ and $t_2$ means were 5.97 (*Sd* = 2.80) and 5.95 (*Sd* = 2.66).

Using Equation A.1 from Appendix 1, we can compute $d_{PPC}$ as

$$d_{PPC} = \frac{\left(M_{\text{post;exp}} - M_{\text{post;ctrl}}\right) - \left(M_{\text{pre;exp}} - M_{\text{pre;ctrl}}\right)}{S_{\text{pre}}} =$$

[(7.52 – 5.95) – (6.28 – 5.97)] / 2.683 = 1.26 / 2.68 = .471.

In order to apply equation A.2 from Appendix 1, first we need to compute the variance of the pre-post differences. This information is not available in the manuscript, but it can be computed it from the pre- and post- variances and the pre-post covariance ($r_{\text{pooled}}$ = .818, pooled covariance = 5.93),

$S_{\text{dif}} = \text{sqrt}(Var_{\text{pre}} + Var_{\text{post}} - 2 \times Cov_{\text{pre,post}}) = \text{sqrt}(7.17 + 7.31 – 2 \times 5.93) = 1.622$

Now $\hat{\omega}^2$ can be computed as

$$\omega^2 = \frac{d_{\text{PPC}}^2 \; n_{\text{exp}} \; n_{\text{ctrl}} \; S_{\text{pre}}^2 - \left(n_{\text{exp}} + n_{\text{ctrl}}\right) S_{\text{dif}}^2}{d_{\text{PPC}}^2 \; n_{\text{exp}} \; n_{\text{ctrl}} \; S_{\text{pre}}^2 + \left(n_{\text{exp}} + n_{\text{ctrl}}\right)\left(4n_{\text{exp}} - 1\right) S_{\text{dif}}^2} =$$

$[471^2 \times 120 \times 66 \times 7.17 - (120+66) \times 1.622^2]$ /

$[471^2 \times 120 \times 66 \times 7.17 + (120+66) \times (4 \times 120-1) \times 1.622]$ =

0.100

Equation 4 can be applied now to estimate the net percentage of changes:

*Net percentage of changes* $\approx B_0 + B_1 \times \hat{\omega}^2 = 2.6 + 152 \times .100 \approx$ 17.86%.

## APPENDIX 3

### Effect of measurement error on the relation between ABC and IBC

In almost all real scenarios, measurement error is expected in any observed variable. However, we have not considered measurement error in our study for a simple reason: it has no effect on the relation between ABC and IBC. Figure A.2 illustrates this idea. It represents the relation between ABC and IBC for three different degrees of measurement error. In the left panel, the pre and post variables are measured without error –as is the case in our simulations. In the center and right panels, the percentage of variance in pre and post due to measurement error is 20% and 40% respectively.

**Figure A.2.** Relation between average-based change (horizontal axis) and individual-based change (vertical axis), as a function of the proportion of variance due to measurement error. Note: Data based on *SID* with *n* = 100, normally distributed scores and pre-post correlation of *r*=.9. Similar results are found with the rest of conditions in the study.

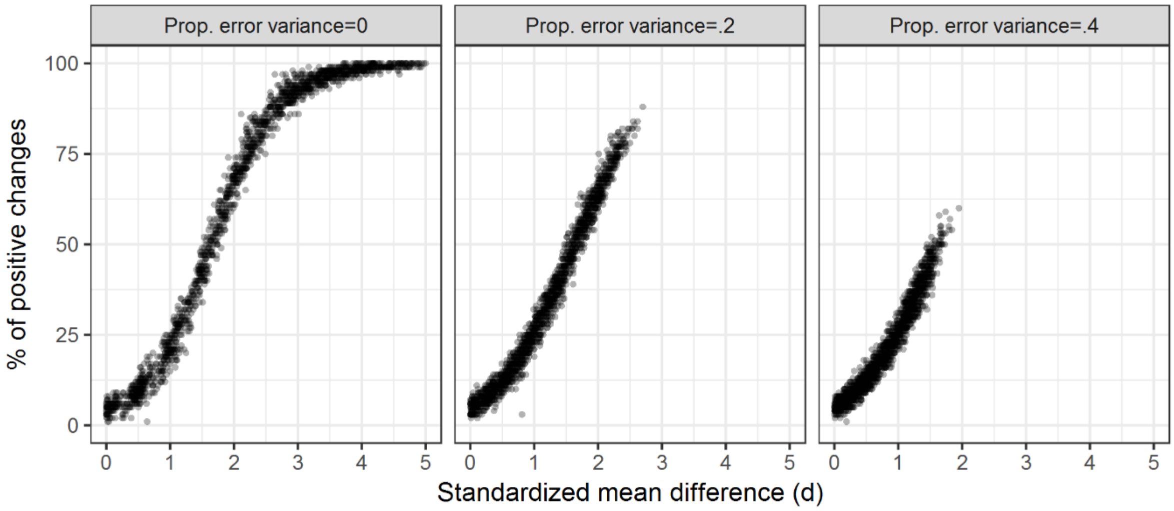

The main consequence of measurement error in a pre-post design is an attenuation of the observed effect size: the *observed* pre-post difference will always be closer to zero than the *true* difference. Higher error variance leads to larger attenuation. Because both ABC and IBC are computed from the same variables, this attenuation affects both types of statistics equally. Therefore, the relation between them remains invariant: any observed ABC corresponds to the same predicted value of IBC, although the range of observed effect size will decrease under both approaches with higher proportions of error variance.
